# Supplementary material for: Newly Designed Poxviral Promoters to Improve Immunogenicity and Efficacy of MVA-NP Candidate Vaccines against Lethal Influenza Virus Infection in Mice
Source: Pathogens. 2023 Jun 23;12(7):867. doi: 10.3390/pathogens12070867 (PMC10383309; doi:10.3390/pathogens12070867)
Supplement: Supplementary file 1 [file pathogens-12-00867-s001.zip › pathogens-2412352-supplementary.pdf]

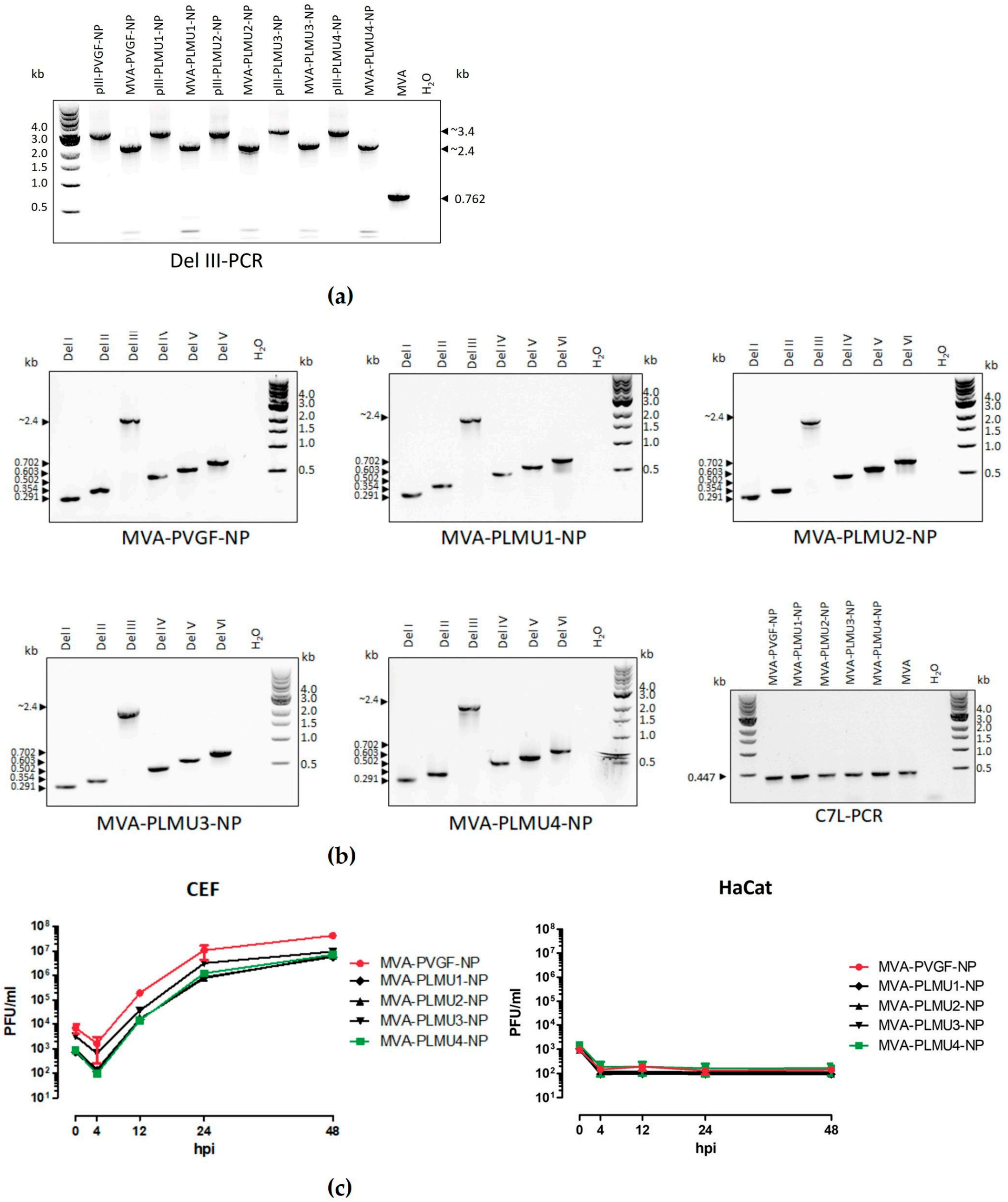

Figure S1: Virological characterization of MVA-PVGF-NP, MVA-LMU1-NP, MVA-LMU2-NP, MVA-LMU3-NP, and MVA-LMU4-NP. (a) Genetic integrity of MVA-PVGF-NP, MVA-LMU1-NP, MVA-LMU2-NP, MVA-LMU3-NP, and MVA-LMU4-NP. PCR analysis of genomic viral DNA confirmed stable insertion of the influenza A virus (IAV) nucleoprotein (NP) sequence into deletion site III of the MVA genome. The intragenomic deletion of the marker gene mCherry during plaque purification was revealed by amplification of a PCR product with the expected molecular weight (~2.4 kb) from genomic DNA compared to the large product amplified from pIIIred-PVGF-NP, pIIIred-PLMU1-NP, pIIIred-PLMU2-NP, pIIIred-PLMU3-NP, and pIIIred-PLMU4-NP plasmid DNA template. The deletion III site-specific oligonucleotide primers amplified a characteristic 0.762 kb DNA fragment from genomic, non-recombinant MVA DNA. **(b)** Genetic stability of MVA-PVGF-NP, MVA-LMU1-NP, MVA-LMU2-NP, MVA-LMU3-NP, and MVA-LMU4-NP. PCR analysis of genomic viral DNA targeting the six major deletion sites or the C7L gene locus of MVA confirmed genetic stability of the recombinant viruses. **(c)** Multiple-step growth analysis of recombinant MVA-PVGF-NP, MVA-LMU1-NP, MVA-LMU2-NP, MVA-LMU3-NP, and MVA-LMU4-NP. Cells were infected at a multiplicity of infection (MOI) of 0.05 with MVA-PVGF-NP, MVA-LMU1-NP, MVA-

LMU2-NP, MVA-LMU3-NP, or MVA-LMU4-NP, and collected at the indicated time-points. Titration was performed on CEF cells and plaque-forming units (PFU) were determined. The recombinant viruses could be efficiently amplified on CEF cells (left panel) but failed to productively grow on the human HaCat cells (right panel).
